# New Species of the Genus *Curvularia*: *C. tamilnaduensis* and *C. coimbatorensis* from Fungal Keratitis Cases in South India

**DOI:** 10.3390/pathogens9010009

**Published:** 2019-12-20

**Authors:** Noémi Kiss, Mónika Homa, Palanisamy Manikandan, Arumugam Mythili, Krisztina Krizsán, Rajaraman Revathi, Mónika Varga, Tamás Papp, Csaba Vágvölgyi, László Kredics, Sándor Kocsubé

**Affiliations:** 1 Department of Microbiology, Faculty of Science and Informatics, University of Szeged, 6726 Szeged, Hungary; kissnoemi621@gmail.com (N.K.); homamoni@gmail.com (M.H.); varga.j.monika@gmail.com (M.V.); pappt@bio.u-szeged.hu (T.P.); csaba@bio.u-szeged.hu (C.V.); 2 MTA-SZTE “Lendület” Fungal Pathogenicity Mechanisms Research Group, 6726 Szeged, Hungary; 3 Department of Medical Laboratory Sciences, College of Applied Medical Sciences, Majmaah University, Al Majmaah 11952, Saudi Arabia; manikandanpalanisamy@gmail.com; 4 Greenlink Analytical and Research Laboratory India Private Ltd., Coimbatore, Tamil Nadu 641014, India; 5 Department of Microbiology, Dr. G.R. Damodaran College of Science, Coimbatore, Tamil Nadu 641014, India; mythilia1689@gmail.com; 6 Synthetic and Systems Biology Unit, Institute of Biochemistry, Biological Research Centre, Hungarian Academy of Sciences, 6726 Szeged, Hungary; krizsank@gmail.com; 7 Aravind Eye Hospital and Postgraduate Institute of Ophthalmology, Coimbatore, Tamil Nadu 641014, India; revathi@aravind.org

**Keywords:** *Curvularia*, keratitis, taxonomy, antifungal susceptibility, *Curvularia coimbatorensis*, *Curvularia tamilnaduensis*

## Abstract

Members of the genus *Curvularia* are melanin-producing dematiaceous fungi of increasing clinical importance as causal agents of both local and invasive infections. This study contributes to the taxonomical and clinical knowledge of this genus by describing two new *Curvularia* species based on isolates from corneal scrapings of South Indian fungal keratitis patients. The phylogeny of the genus was updated based on three phylogenetic markers: the internal transcribed spacer (ITS) region of the ribosomal RNA gene cluster as well as fragments of the glyceraldehyde-3-phosphate dehydrogenase (*gpdh*) and translation elongation factor 1-α (*tef1α*) genes. The maximum likelihood phylogenetic tree constructed from the alignment of the three concatenated loci revealed that the examined isolates are representing two new, yet undescribed, *Curvularia* species. Examination of colony and microscopic morphology revealed differences between the two species as well as between the new species and their close relatives. The new species were formally described as *Curvularia tamilnaduensis* N. Kiss & S. Kocsubé sp. nov. and *Curvularia coimbatorensis* N. Kiss & S. Kocsubé sp. nov. Antifungal susceptibility testing by the broth microdilution method of CLSI (Clinical & Laboratory Standards Institute) revealed that the type strain of *C. coimbatorensis* is less susceptible to a series of antifungals than the *C. tamilnaduensis* strains.

## 1. Introduction

The fungal genus *Curvularia* (Ascomycota, Pleosporales, Pleosporaceae) comprises of dematiaceous, melanin-producing molds with various lifestyles including saprophytism, plant endophytism [1], plant parasitism [2], and human pathogenicity [3].

The genus-level identification of *Curvularia* was performed traditionally by the examination of pigmentation, as well as the morphology of the septate conidia and hyphae [3]. The first sequence-based species-level identification attempts targeted the internal transcribed spacer (ITS) region of the ribosomal RNA gene cluster, which alone, however, proved inappropriate, either for the purposes of exact diagnosis [4] or for the phylogenetic resolution of the genus and the clarification of its relationship to the closely related genera *Bipolaris*, *Cochliobolus*, and *Drechslera* [3]. Multilocus sequence typing (MLST) involving fragments of the nuclear ribosomal large subunit RNA (LSU) as well as the glyceraldehyde-3-phosphate dehydrogenase (*gpdh*) and translation elongation factor 1-α (*tef1a*) genes in addition to ITS had resulted in the recently accepted phylogenetic concept of the genus *Curvularia* [5], which was applied in more recent works [6,7,8]. Recently, the genus involves more than 100 described species, which can be divided into six clades (americana, eragrostidis, hominis, lunata, spicifera, and trifolii) according to Madrid et al. [7] based on MLST of four loci (ITS, LSU, *gpdh*, and the RNA polymerase II subunit *rpb2*).

Krizsán et al. [3] reviewed the clinical importance of the genus *Curvularia*, and identified *Curvularia australiensis*, *Curvularia geniculata*, *Curvularia hawaiiensis*, *Curvularia lunata*, * Curvularia pallescens*, and *Curvularia spicifera* as the species most frequently isolated from clinical samples. Further members of the genus with confirmed clinical relevance include *Curvularia americana*, *Curvularia chlamydospora*, *Curvularia hominis*, *Curvularia muehlenbeckiae*, *Curvularia pseudolunata* [7], *Curvularia brachyspora* [9], *Curvularia senegalensis* [10,11], *Curvularia clavata* [12], *Curvularia tuberculata* [13], and *Curvularia inaequalis* [14,15,16]. A *Curvularia* infection in humans is designated as curvulariosis, a subtype of phaeohyphomycoses (i.e., fungal infections caused by dematiaceous fungi) [3]. The resulting diseases include deep and disseminated infections [3,17,18,19], infections complicating peritoneal dialysis [14,20,21], respiratory infections including sinusitis and bronchopulmonary mycosis [3,10,22], urinary tract infections [23], as well as localized infections affecting the skin, nail [4,24,25], and the eye. Among eye infections, the involvement of *Curvularia* spp. is most frequent in keratitis—a suppurative, ulcerative disease of the cornea, but endophthalmitis and chronic dacryocystitis cases have also been reported [3,26].

In this study, we describe two new species of the genus *Curvularia*, the type strains of which were isolated from corneal scraping samples derived from South Indian patients diagnosed with fungal keratitis.

## 2. Results

### 2.1. Strain Selection and Case Details

About two thirds of the dematiaceous fungi isolated from corneal ulcers in the Aravind Eye Hospital, Coimbatore, Tamil Nadu, India belong to the genus *Curvularia* (unpublished data). The four strains involved in this study were selected retrospectively based on the inability of reliable species-level identification of some *Curvularia* isolates by ITS sequence analysis. Details available of the cases are presented in Table 1. All four patients were diagnosed with fungal corneal ulcer. The corneal scrapings from the ulcers were in all cases positive for fungal filaments in direct microscopy (both 10% KOH and Gram staining). None of the cases had a history of contact lens wear. History of falling dust (2) and mud (1) into the eye was recorded as predisposing factors. Based on the typical clinical picture and the KOH report, topical antifungal therapy was started with natamycin (5% suspension) and econazole drops (2%) every half an hour, along with homatropine (1%) administered three times a day. Unfortunately, the patients were lost to follow up after one or two visits.

### 2.2. Updated Phylogeny of the Genus Curvularia

Table 2 shows the strains and sequences involved in the phylogenetic analysis of the genus *Curvularia*, including four isolates derived from cases of fungal keratitis diagnosed and treated in the Aravind Eye Hospital, Coimbatore, Tamil Nadu, India. The *tef1α* dataset consisted of 902 characters of nucleotide alignment without binary characters. The *gpdh* dataset contained 684 characters with 601 characters of nucleotide alignment and 63 binary characters derived from indel coding. The length of the ITS alignment was 1193 characters long, containing 896 bp of nucleotide data and 297 binary characters.

**Figure 1:**
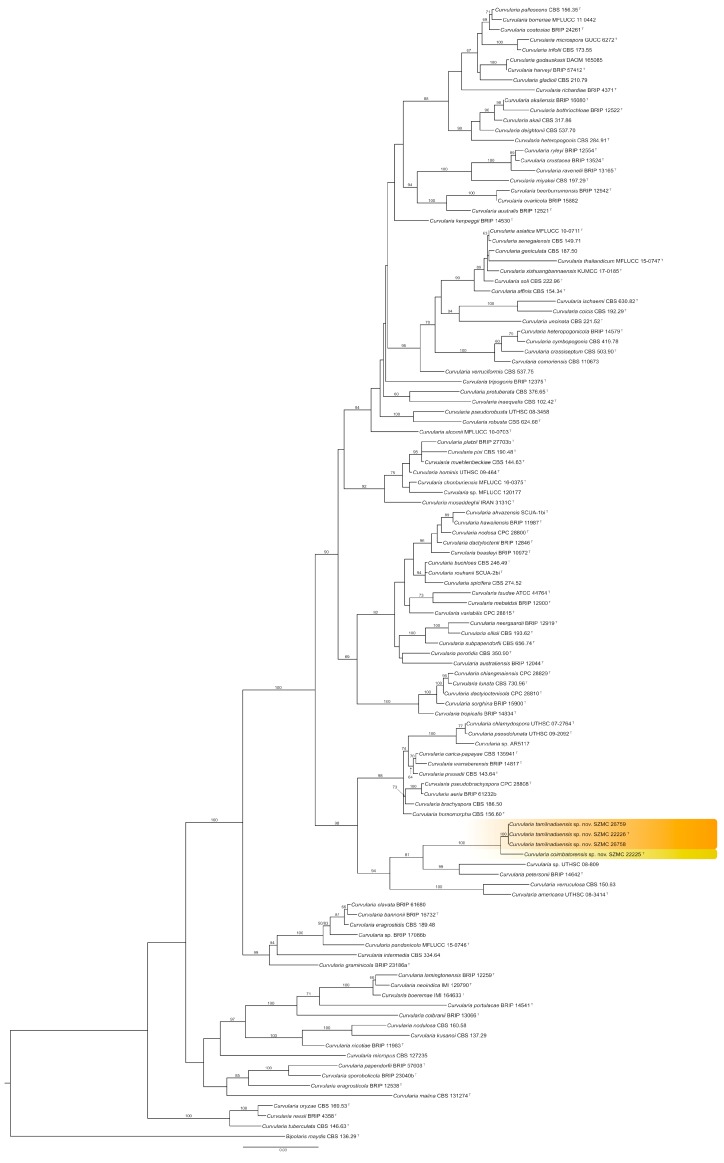
Maximum likelihood phylogeny of the genus *Curvularia* inferred from the concatenated internal transcribed spacer (ITS), translation elongation factor 1-α (*tef1a*), and glyceraldehyde-3-phosphate dehydrogenase (*gpdh*) sequences. The isolates examined in this study are shown as the new species *Curvularia tamilnaduensis* and *Curvularia coimbatorensis* (highlighted in color). Sequences of the reference *Curvularia* strains were collected from the GenBank Nucleotide database (Table 1). Bootstrap support values greater than 60% are shown above the branches. *Bipolaris maydis* CBS 136.29 was used to root the tree. Abbreviations of culture collections: BRIP: Plant Pathology Herbarium, Queensland, Australia; CBS: Westerdijk Fungal Biodiversity Institute culture collection, The Netherlands; CPC: Cultures of Pedro Crous, housed at Westerdijk Fungal Biodiversity Institute; DAOM: Canadian National Mycological Herbarium, Ottawa, Canada; GUCC: Guizhou University Culture Collection, Guizhou, China; IMI: CABI Bioscience, Eggham, UK; IRAN: Iranian Fungal Culture Collection, Iranian Research Institute of Plant Protection, Tehran, Iran; KUMCC: Culture Collection of Kunming Institute of Botany, Kunming, China; MFLUCC: Mae Fah Luang Culture Collection, Chiang Rai, Thailand; SCUA: Collection of Fungal Cultures, Department of Plant Protection, Shahid Chamran University of Ahvaz, Iran; SZMC: Szeged Microbiology Collection, Szeged, Hungary; UTHSC: University of Tennessee Health Science Center, Memphis, USA. **^T^**: type strain.

On the phylograms obtained from each of the three loci, the four keratitis isolates of this study were resolved as two new species with over 80% of confidence values (data not shown), one of them represented by the single isolate SZMC 22225, while the other one by isolates SZMC 22226, SZMC 26758, and SZMC 26758. As the individual inferences were largely congruent, the three loci were concatenated and partitioned. The phylogenetic tree obtained from the concatenated dataset is shown in Figure 1.

### 2.3. Taxonomy and Related Information

**Figure 2:**
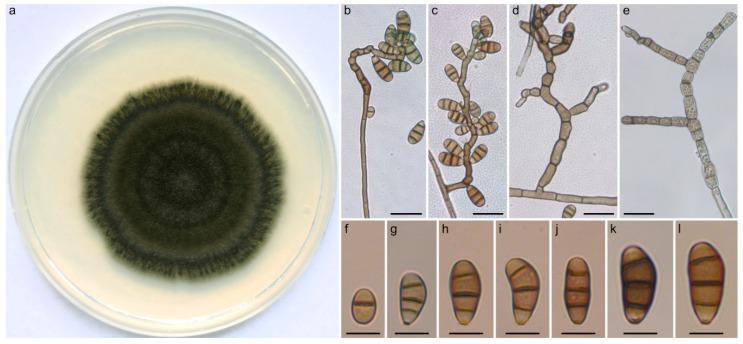
Morphological features of *Curvularia coimbatorensis* SZMC 2225. (**a**) Colony morphology on PDA (potato dextrose agar) medium after 7 days at 25 °C; (**b**,**c**) conidiophores with septate conidia; (**d**) branching conidiophores; (**e**) swollen cells; (**f**–**l**) septate conidia. Scale bars: (**b**–**e**) 20 µm; (**f**–**l**) 10 µm.

*Curvularia coimbatorensis* N. Kiss & S. Kocsubé sp. nov. (Figure 2). MycoBank accession number: MB 833656. The etymology is referring to the city in Tamil Nadu, South India where the type strain was isolated.

Vegetative hyphae septate, subhyaline to brown, branched, smooth, 3–4 µm in width. Colonies on PDA reaching approximately 4–6 cm in diameter after 7 days at 25 °C, surface funiculose, margin fimbriate, olivaceous black to olivaceous grey, velutinous with sparse aerial mycelium. Conidiophores erect, often branched, in most cases uniformly brown, sometimes pale brown at apex, seminematous, septate, flexuous, in most cases geniculate towards the apex, up to 210 µm long, 3–4 µm wide, basal cells sometimes swollen. Conidiogenous cells integrated, terminal, or intercalary with sympodial proliferation, smooth, brown, mono- or polytretic. Chlamydospores not observed. Conidia ellipsoidal to clavate to obovoid, asymmetrical with paler end cells, usually curved at the third cell from the base, (13-)16–18(-23) × (7-)8–9(-10) µm, 3-distoseptate, hila slightly protuberant, thickened and darkened.

Specimens examined: India, Coimbatore, human corneal scraping from corneal ulcer, 2012, (holotype: freeze dried culture specimen in the Szeged Microbiological Collection (SZMC) at the Department of Microbiology, Faculty of Science and Informatics, University of Szeged, Hungary, SZMC 22225, includes ex-type culture).

**Figure 3:**
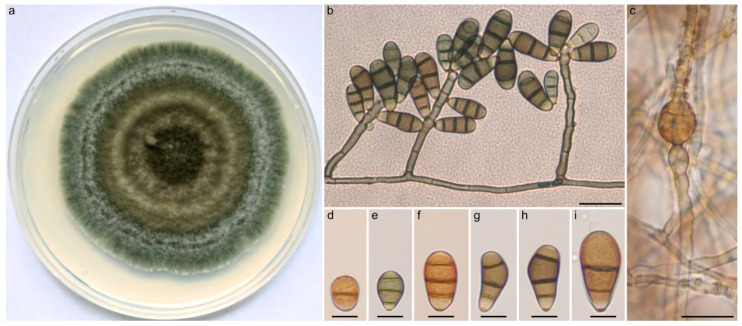
Morphological features of *Curvularia tamilnaduensis* SZMC 2226. (**a**) Colony morphology on PDA medium after 7 days at 25 °C; (**b**) conidiophores with septate conidia; (**c**) subglobose intercalary chlamydospore; (**d**–**f**) septate conidia. Scale bars: (**b**,**c**) 20 µm; (**d**–**i**) 10 µm.

*Curvularia tamilnaduensis* N. Kiss & S. Kocsubé sp. nov. (Figure 3). MycoBank accession number: MB 833657. The etymology is referring to the state of South India where the type strain and the other two examined strains were isolated.

Vegetative hyphae septate, subhyaline to brown, branched, smooth walled, but often heavily asperulate, 2–3 µm in width. Colonies on PDA reaching approximately 6–7 cm in diameter after 7 days at 25 °C, surface lanose, aerial mycelium abundant, margin fimbriate, olivaceous green. Conidiophores erect, usually unbranched, in most cases uniformly brown, sometimes with paler tip, seminematous, septate, slightly flexuous, rarely geniculate towards the apex, up to 125 µm long, 2.5–4 µm wide. Conidiogenous cells integrated, terminal or intercalary, smooth, pale brown to brown, mono- or polytretic, proliferating sympodially. Chlamydospores present, subglobose, terminal and intercalary, 8–22 µm in diameter. *Conidia* ellipsoidal to clavate to obovoid, asymmetrical with paler basal and apical cells, usually curved at the third cell from the base which is darker than the other cells, (15-)20–23(-28) × (7-)8–10(-11) µm, (2-)3-distoseptate with non-protuberant, thickened, and darkened hila.

Specimens examined: India, Coimbatore, human corneal scraping from corneal ulcer, 2013, (holotype: freeze dried culture specimen in the Szeged Microbiological Collection (SZMC) at the Department of Microbiology, Faculty of Science and Informatics, University of Szeged, Hungary, SZMC 22226, includes ex-type culture); India, Coimbatore, human corneal scraping from corneal ulcer, 2011, (SZMC 26758); India, Coimbatore, human corneal scraping from corneal ulcer, 2011–2013, (SZMC 26759).

### 2.4. Antifungal Susceptibilities of Curvularia Strains Isolated from Fungal Keratitis

The minimum inhibitory concentrations (MIC) of nine antifungal agents towards *C. coimbatorensis* SZMC 22225, *C. tamilnaduensis* SZMC 22226, SZMC 26758, and SZMC 26759, as well as the type strains of *C. australiensis* (CBS 172.57), *C. hawaiiensis* (CBS 173.57), and *C. spicifera* (CBS 274.52) are shown in Table 3. The MIC of natamycin was 2 µg mL^−1^ for both new species and all other strains tested, while substantial differences between them could be observed in the case of clotrimazole, econazole, miconazole, and terbinafine, with the type strain of *C. coimbatorensis* having 4, 8, 4, and 4–8 times higher values, respectively. Among the tested isolates, the type strain of *C. spicifera* proved to be the less susceptible to clotrimazole, econazole, fluconazole, ketoconazole, and miconazole. Notable strain-to-strain variations between the *C. tamilnaduensis* strains could be observed only in the case of itraconazole and ketoconazole with detected MIC ranges of 0.03–0.25 and 0.06–0.25, respectively.

## 3. Discussion

The phylogenetic tree obtained from the concatenated dataset of three loci presents an update about the phylogeny of *Curvularia*, which is mostly in agreement with the recently published phylogenies of this genus (Figure 1). *C. ischaemi* formed a clade with *C. coicis*, which is in contradiction with the results of Tan et al. [8] and Tibpromma et al. [27], where *C. ischaemi* formed a sister clade to *C. gladioli*, but in agreement with the phylogram obtained by Madrid et al. [7] and Manamgoda et al. [28]. Our analysis placed *C. perotidis* as a sister clade to *C. australiensis,* however, other studies [7,8,27,29] suggested that this species is closer to *C. spicifera*. The placement of *C. variabilis* was also different from previously published articles [8,29]. According to the analyses of Tan et al. [8] and Marin-Felix et al. [29], *C. variabilis* forms a clade with *C. hawaiiensis*, *C. nodosa*, *C. dactyloctenicola*, and *C. beasleyi*, however, in this study we found *C. variabilis* as a sister clade of *C. tsudae* and *C. mebaldsii*. The same authors found *C. tripogonis*, *C. pseudorobusta*, *C. robusta*, *C. alcornii*, *C. protuberata*, and *C. inaequalis* as members of two distinct monophyletic clades, while our results indicate that these species are closely related and paraphyletic, however, none of the topologies have strong statistical supports. The observed slight differences between the previous inferences and our analyses did not affect the validity of any of the previously described species, and some of them might be the result of the slightly broader taxon sampling.

One of the newly described species, *C. coimbatorensis* is only known from the type specimen isolated from corneal ulcer. Phylogenetic analysis based on three loci placed *C. coimbatorensis* as a sister clade to the other newly described species *C. tamilnaduensis*. The two species are closely related, but can be distinguished by *tef1a*, *gpdh*, and ITS sequences, with percentage identities of 99%, 98%, and 99%, respectively. *C. petersonii* [8] is also closely related and can be distinguished by all three loci (98% in *tef1a*, 93% in *gpdh* and 96% in ITS). *C. coimbatorensis* differs from *C. tamilnaduensis* in colony morphology, the lack of chlamydospores, and the size of conidia. *C. petersonii* is very similar in colony morphology, however, has significantly shorter (up to 110 µm) and only slightly geniculate conidiophores bearing narrower (5-)5.5–6(-7) conidia [8]. *C. coimbatorensis* has longer conidiophores.

The phylogenetic analysis based on three loci placed the other newly described species, *C. tamilnaduensis* as a sister clade to the recently described species *C. petersonii*. *C. tamilnaduensis* can be reliably distinguished from the ex-type of *C. petersonii* by *tef1a*, *gpdh* and ITS sequences with percentage identities of 99%, 95%, and 96%, respectively. The two species also differ by morphology, as *C. petersonii* has not been reported to produce chlamydospores and has different conidial dimensions (17–19 × 5.5–6) [8]. *C. americana* [7] and *C. verruculosa* [30] are also related species with considerable amount of genetic distances and none of these species have been reported before to have chlamydopores. *C. americana* has 4(-5)-distoseptate and wider (7–15 µm) conidia, while *C. verruculosa* has mostly 3-distoseptate conidia, but also wider (12–17 µm) than those of *C. tamilnaduensis*.

The antifungal susceptibilities of the examined strains of *C. coimbatorensis* and *C. tamilnaduensis* to amphotericin B, clotrimazole, econazole, fluconazole, itraconazole, ketoconazole, miconazole, natamycin, and terbinafine were within the MIC ranges reported for other clinically relevant *Curvularia* species in the study of Guarro et al. [11] and the review of Krizsán et al. [3]. The type strain of *C. coimbatorensis* proved to be less susceptible than the strains of *C. tamilnaduensis* to all antifungals except for natamycin. For itraconazole and ketoconazole our results are in agreement with the study of Guarro et al. [11], who reported that amphotericin B, itraconazole, miconazole and ketoconazole are highly effective against a series of *Curvularia* species known from fungal keratitis (*C. brachyspora*, *C. clavata*, *C. geniculata*, *C. lunata*, *C. pallescens*, *C. senegalensis*, and *C. verruculosa*).

## 4. Materials and Methods

### 4.1. Curvularia Strains, Culture Conditions, and Morphological Examination

The *Curvularia* strains involved in this study derived from corneal scrapings from fungal corneal ulcers of keratitis patients attending the Aravind Eye Hospital and Postgraduate Institute of Ophthalmology, Coimbatore, India. All cases were initially screened by experienced ophthalmologists, and the corneal scrapings were collected following the clinical diagnosis of fungal keratitis. The samples were initially processed microbiologically for the isolation of the causative agents as described earlier [31]. The corneal scrapings of all patients were subjected to Gram stain, Giemsa stain, and 10% KOH wet mount. Culture methods involved direct inoculation of specimens onto 5% sheep blood agar, chocolate agar, non-nutrient agar, potato dextrose agar, thioglycolate broth, and brain–heart infusion broth. The microbial cultures were considered positive only if the growth of the same organism was demonstrated on two or more solid media, or there was confluent growth at the site of inoculation on one solid medium with consistent direct microscopic findings. The isolates were deposited in the Szeged Microbiology Collection (SZMC, Szeged, Hungary) under the accession numbers SZMC 22225, SZMC 22226, SZMC 26758, and SZMC 26759. Colony morphology of the isolates was examined on PDA (BioLab, Budapest, Hungary) medium after 7 days of incubation at 25 °C under normal day/night light conditions. Micromorphological characters were examined with a Leica DMI 4000B (Leica, Wetzlar, Germany) microscope equipped with a Leica DFC 295 camera. Microscopic features were examined in lactic acid (100% *v*/*v*) on glass slides. Conidiophores were studied in the same mounting fluid with the transparent tape method. Conidiophores and conidia were measured using the software ImageJ v2.52a (National Institute of Mental Health, Bethesda, MD, USA). Size ranges of the conidia were derived from 50 measurements. Lengths and widths are given as (minimum value) mean size minus SD-mean size plus SD (maximum value).

### 4.2. DNA Extraction, Amplification, Sequencing, and Phylogenetic Analysis

Genomic DNA was isolated from the examined *Curvularia* strains SZMC 22225, SZMC 22226, SZMC 26758, and SZMC 26759 with the Masterpure™ Yeast DNA Purification Kit (Epicentre Biotechnologies, Madison, WI, USA) according to the manufacturer’s instructions. Fragments of *tef1a* and *gpdh* were amplified as described previously [5,32,33]. The ITS region of the ribosomal RNA gene cluster was amplified according to White et al. [34]. Sequencing of the amplicons was carried out on a 3500 Genetic Analyzer (Thermo Fisher Scientific, Waltham, MA, USA) by the sequencing service of the Biological Research Centre, Szeged, Hungary. Resulting sequences were deposited in the GenBank Nucleotide database (www.ncbi.nlm.nih.gov) under the accession numbers shown in Table 2.

Sequences of the four clinical isolates were aligned with publicly available sequences of 108 previously described *Curvularia* species, as well as *Bipolaris maydis* as the outgroup (Table 2). Phylogenetic analyses were conducted using three loci (*tef1α*, *gpdh* and ITS). Sequences of all three loci were aligned with the phylogeny-aware sequence alignment tool Canopy v0.1.4 using RAxML as tree estimator and PRANK [35] with the -F option as the aligner with 10 iterations and seed decomposition strategy. Alignments of the three loci were concatenated and partitioned by region. The *tef1α* sequences formed one partition while in the case of *gpdh* sequences the dataset was partitioned to exons and introns. The ITS dataset was divided to rDNA and ITS1-ITS2 regions. Alignments of *gpdh* and ITS datasets contained high number of indels with important phylogenetic signal, therefore gaps were coded as absence/presence characters by SequenceMatrix v1.8 [36] using the simple indel coding algorithm [37]. The two indel matrices were concatenated and added as a single partition to the dataset. Maximum likelihood analysis was performed using RAxML-NG v0.9.0 [38] under the GTR model with gamma-distributed rate heterogeneity using empirical base frequencies. As indel-based datasets do not contain constant sites, the ascertainment bias correction described by Lewis [39] was used for this partition. Statistical support of the best ML tree was obtained with 1000 thorough bootstrap replicates.

### 4.3. Antifungal Susceptibility Testing

In vitro antifungal susceptibility tests were carried out according to the CLSI M38-A2 broth microdilution method [40]. Nine antifungal agents: amphotericin B, clotrimazole, econazole, fluconazole, itraconazole, ketoconazole, miconazole, natamycin and terbinafine (Sigma-Aldrich, Budapest, Hungary) were examined. Microtiter plates were incubated at 35 °C for 72 h. Plates were evaluated both spectrophotometrically with a Spectrostar Nano microplate reader (BMG Labtech, Ortenberg, Germany) and by visual examination. 

## 5. Conclusions

The present study demonstrates, that although the phylogeny of the genus *Curvularia* is resolved and well established, further expansion can be expected both in the list of described *Curvularia* species and in the known spectrum of clinically relevant members of the genus. The collection of further keratitis isolates from the genus *Curvularia* and gaining data about their antifungal susceptibilities are therefore tasks of increasing importance. Furthermore, comparing the infectivity of various *Curvularia* species causing keratitis—including the recently described ones—in animal keratitis models would be an intriguing topic for future research.

## Figures and Tables

**Table 1:** Case details of the fungal keratitis infections.

Strain	Age	Sex	Clinical Diagnosis	Corneal Scraping	Therapy	Outcome
SZMC 22225	80	Male	Fungal corneal ulcer	11 July 2012	NAT, ECZ, HTR	Lost to follow up after two visits
SZMC 22226	66	Male	Fungal corneal ulcer	2 March 2013	NAT, ECZ, HTR	Lost to follow up after one visit
SZMC 26758	40	Male	Fungal corneal ulcer	21 March 2011	NAT, ECZ, HTR	Lost to follow up after one visit
SZMC 26759	NA	NA	Fungal corneal ulcer	NA	NA	Lost to follow up

NAT: natamycin (5%); ECZ: econazole (2%), HTR: homatropine (1%); NA: data not available.

**Table 2:** Sequences used for the phylogenetic analysis.

*Curvularia* Species	Strain	GenBank Accession Number		
		ITS	*tef1a*	*gpdh*
*Bipolaris maydis*	CBS 136.29 ^T^	KJ909780	KM093794	KM034846
*Curvularia aeria*	BRIP 61232b	KX139029	KU552155	KU552162
*Curvularia affinis*	CBS 154.34 ^T^	KJ909782	KM196566	KM230401
*Curvularia ahvazensis*	SCUA-1bi ^T^	KJ415539	MG428686	MG428693
*Curvularia akaii*	CBS 317.86	JX256420	KM196569	KM230402
*Curvularia akaiiensis*	BRIP 16080 ^T^	HE861833	KJ415453	KJ415407
*Curvularia alcornii*	MFLUCC 10-0703 ^T^	JX256424	JX266589	JX276433
*Curvularia americana*	UTHSC 08-3414 ^T^	KJ415540	-	HF565488
*Curvularia asiatica*	MFLUCC 10-0711 ^T^	KJ415541	JX266593	JX276436
*Curvularia australiensis*	BRIP 12044 ^T^	KJ415542	KJ415452	KJ415406
*Curvularia australis*	BRIP 12521 ^T^	MH414892	KJ415451	KJ415405
*Curvularia bannonii*	BRIP 16732 ^T^	MH414894	KJ415450	KJ415404
*Curvularia beasleyi*	BRIP 10972 ^T^	MH414911	MH433654	MH433638
*Curvularia beerburrumensis*	BRIP 12942 ^T^	KP400638	MH433657	MH433634
*Curvularia boeremae*	IMI 164633 ^T^	KJ415543	-	MH433641
*Curvularia borreriae*	MFLUCC 11-0422	KJ922372	KM196571	KP419987
*Curvularia bothriochloae*	BRIP 12522 ^T^	KJ909765	KJ415449	KJ415403
*Curvularia brachyspora*	CBS 186.50	HG778984	KM230405	KM061784
*Curvularia buchloës*	CBS 246.49 ^T^	MF490814	KM196588	KM061789
*Curvularia carica-papayae*	CBS 135941 ^T^	HG779021	-	HG779146
*Curvularia chiangmaiensis*	CPC 28829 ^T^	MH275055	MF490857	MF490836
*Curvularia chlamydospora*	UTHSC 07-2764 ^T^	KU552205	-	HG779151
*Curvularia chonburiensis*	MFLUCC 16-0375 ^T^	MH414897	-	MH412747
*Curvularia clavata*	BRIP 61680b	AF081447	KU552159	KU552167
*Curvularia coatesiae*	BRIP 24261 ^T^	MH414898	MH433659	MH433636
*Curvularia coicis*	CBS 192.29 ^T^	LT631357	JN601006	AF081410
*Curvularia colbranii*	BRIP 13066 ^T^	LT631310	MH433660	MH433642
*Curvularia comoriensis*	CBS 110673	KJ415544	-	LT715841
*Curvularia crassiseptum*	CBS 503.90 ^T^	HG778985	-	LT715882
*Curvularia crustacea*	BRIP 13524 ^T^	MF490815	KJ415448	KJ415402
*Curvularia cymbopogonis*	CBS 419.78	KJ415545	-	HG779129
*Curvularia dactyloctenicola*	CPC 28810 ^T^	LT631356	MF490858	MF490837
*Curvularia dactyloctenii*	BRIP 12846 ^T^	JN192375	KJ415447	KJ415401
*Curvularia deightonii*	CBS 537.70	MH414899	-	LT715839
*Curvularia ellisii*	CBS 193.62 ^T^	HG778986	JN601007	JN600963
*Curvularia eragrosticola*	BRIP 12538 ^T^	KJ909781	MH433661	MH433643
*Curvularia eragrostidis*	CBS 189.48	HG778987	-	HG779154
*Curvularia geniculata*	CBS 187.50	JN192376	KM230410	KM083609
*Curvularia gladioli*	CBS 210.79	KJ415546	-	HG779123
*Curvularia graminicola*	BRIP 23186a ^T^	KJ415547	JN601008	JN600964
*Curvularia harveyi*	BRIP 57412 ^T^	KJ415548	KJ415446	KJ415400
*Curvularia hawaiiensis*	BRIP 11987 ^T^	KJ415549	KJ415445	KJ415399
*Curvularia heteropogonicola*	BRIP 14579 ^T^	HG779011	KJ415444	KJ415398
*Curvularia heteropogonis*	CBS 284.91 ^T^	JN192380	JN601013	JN600969
*Curvularia hominis*	CBS 136985 ^T^	KJ922375	-	HG779106
*Curvularia homomorpha*	CBS 156.60 ^T^	HG778991	JN601014	JN600970
*Curvularia inaequalis*	CBS 102.42 ^T^	MH861533	KM196574	KM061787
*Curvularia intermedia*	CBS 334.64	MH414900	-	HG779155
*Curvularia ischaemi*	CBS 630.82 ^T^	MH855025	-	LT715790
*Curvularia kenpeggii*	BRIP 14530 ^T^	MH414901	MH433662	MH433644
*Curvularia kusanoi*	CBS 137.29	JX256429	JN601016	LT715862
*Curvularia lamingtonensis*	BRIP 12259 ^T^	JF812154	MH433663	MH433645
*Curvularia lunata*	CBS 730.96 ^T^	MH414902	JX266596	JX276441
*Curvularia malina*	CBS 131274 ^T^	HE792934	KR493095	KP153179
*Curvularia mebaldsii*	BRIP 12900 ^T^	MF139088	MH433664	MH433647
*Curvularia micropus*	CBS 127235	KJ909770	-	LT715859
*Curvularia microspora*	GUCC6272 ^T^	MG846737	MF139115	MF139106
*Curvularia miyakei*	CBS 197.29 ^T^	KP400647	KM196568	KM083611
*Curvularia mosaddeghii*	IRAN 3131C ^T^	KJ415550	MH392152	MH392155
*Curvularia muehlenbeckiae*	CBS 144.63 ^T^	MH414910	KM196578	KP419996
*Curvularia neergaardii*	BRIP 12919 ^T^	KJ415551	KJ415443	KJ415397
*Curvularia neoindica*	IMI 129790 ^T^	MF490816	MH433667	MH433649
*Curvularia nicotiae*	BRIP 11983 ^T^	JN601033	KJ415442	KJ415396
*Curvularia nodosa*	CPC 28800 ^T^	KP400650	MF490859	MF490838
*Curvularia nodulosa*	CBS 160.58	JN192384	JN601019	JN600975
*Curvularia oryzae*	CBS 169.53 ^T^	KJ922380	KM196590	KP645344
*Curvularia ovariicola*	CBS 470.90 ^T^	MH275056	JN601020	JN600976
*Curvularia pallescens*	CBS 156.35 ^T^	KJ415552	KM196570	KM083606
*Curvularia pandanicola*	MFLUCC 15-0746 ^T^	HG778995	MH412763	MH412748
*Curvularia papendorfii*	CBS 308.67 ^T^	MH414905	KJ415441	KJ415395
*Curvularia perotidis*	CBS 350.90 ^T^	KY905678	KM230407	HG779138
*Curvularia petersonii*	BRIP 14642 ^T^	MH414906	MH433668	MH433650
*Curvularia pisi*	CBS 190.48 ^T^	KJ415553	KY905697	KY905690
*Curvularia platzii*	BRIP 27703b ^T^	KJ922373	MH433669	MH433651
*Curvularia portulacae*	BRIP 14541 ^T^	KJ922376	KJ415440	KJ415393
*Curvularia prasadii*	CBS 143.64 ^T^	MF490819	KM230408	KM061785
*Curvularia protuberata*	CBS 376.65 ^T^	HE861842	KM196576	KM083605
*Curvularia pseudobrachyspora*	CPC 28808 ^T^	HE861838	MF490862	MF490841
*Curvularia pseudolunata*	UTHSC 09-2092 ^T^	JN192386	-	HF565459
*Curvularia pseudorobusta*	UTHSC 08-3458	MH414907	-	HF565476
*Curvularia ravenelii*	BRIP 13165 ^T^	KJ415555	JN601024	JN600978
*Curvularia reesii*	BRIP 4358 ^T^	KJ909783	MH433670	MH433637
*Curvularia richardiae*	BRIP 4371 ^T^	KX139030	KJ415438	KJ415391
*Curvularia robusta*	CBS 624.68 ^T^	KJ415556	KM196577	KM083613
*Curvularia rouhanii*	SCUA-2bi-2 ^T^	HG779001	MG428687	MG428694
*Curvularia ryleyi*	BRIP 12554 ^T^	KY905679	KJ415437	KJ415390
*Curvularia senegalensis*	CBS 149.71	KJ415558	-	HG779128
*Curvularia soli*	CBS 222.96 ^T^	MH414904	KY905698	KY905691
*Curvularia sorghina*	BRIP 15900 ^T^	KP400655	KJ415435	KJ415388
*Curvularia* sp.	BRIP 17068b	KP400654	MH433666	MH433648
*Curvularia* sp.	AR5117	HE861826	KP735698	KP645349
*Curvularia* sp.	MFLUCC 120177	JN192387	KP735697	KP645348
*Curvularia* sp.	UTHSC 8809	MH414908	-	HF565477
*Curvularia spicifera*	CBS 274.52	KJ909777	JN601023	JN600979
*Curvularia sporobolicola*	BRIP 23040b ^T^	MH275057	MH433671	MH433652
*Curvularia subpapendorfii*	CBS 656.74 ^T^	HG779023	KM196585	KM061791
*Curvularia thailandicum*	MFLUCC 15-0747 ^T^	JN192388	MH412764	MH412749
*Curvularia trifolii*	CBS 173.55	KJ415559	-	HG779124
*Curvularia tripogonis*	BRIP 12375 ^T^	KC424596	JN601025	JN600980
*Curvularia tropicalis*	BRIP 14834 ^T^	JX256433	KJ415434	KJ415387
*Curvularia tsudae*	ATCC 44764 ^T^	HG779024	KC503940	KC747745
*Curvularia tuberculate*	CBS 146.63 ^T^	MF490822	JX266599	JX276445
*Curvularia uncinate*	CBS 221.52 ^T^	HG779026	-	HG779134
*Curvularia variabilis*	CPC 28815 ^T^	KP400652	MF490865	MF490844
*Curvularia verruciformis*	CBS 537.75	MH414909	-	HG779133
*Curvularia verruculosa*	CBS 150.63	MH275058	KP735695	KP645346
*Curvularia warraberensis*	BRIP 14817 ^T^	AF071338	MH433672	MH433653
*Curvularia xishuangbannaensis*	KUMCC 17-0185 ^T^	KJ909780	MH412765	MH412750
*Curvularia gudauskasii*	DAOM 165085	KX139029	KM093794	AF081393
*Curvularia tamilnaduensis* sp. nov.	SZMC 22226 ^T^ *	**MN628311**	**MN628303**	**MN628307**
	SZMC 26758 *	**MN628308**	**MN628300**	**MN628304**
	SZMC 26759 *	**MN628309**	**MN628301**	**MN628305**
*Curvularia coimbatorensis* sp. nov.	SZMC 22225 ^T^ *	**MN628310**	**MN628302**	**MN628306**

^T^ type strain; * Strains examined during the present study. Sequences derived from the present study are set in bold.

**Table 3:** Antifungal susceptibilities of the *Curvularia coimbatorensis* and *Curvularia tamilnaduensis* strains in comparison with the type strains of *Curvularia australiensis*, *Curvularia hawaiiensis*, and *Curvularia spicifera* determined by the CLSI (Clinical & Laboratory Standards Institute) broth microdilution method (minimum inhibitory concentrations (MIC) values in µg mL^−1^).

Strain	Antifungal Agent								
	AMB	CLT	ECN	FLC	ITC	KTC	MCZ	NTM	TRB
*C. australiensis* CBS 172.57 ^T^	0.25	0.25	0.125	16	0.03	0.25	0.25	2	0.25
*C. hawaiiensis* CBS 173.57 ^T^	0.25	0.06	0.06	4	0.03	0.06	0.125	2	0.25
*C. spicifera* CBS 274.52 ^T^	0.5	4	2	>32	0.25	2	2	2	1
*C. coimbatorensis* SZMC 22225 ^T^	0.5	0.5	1	32	0.25	0.25	1	2	1
*C. tamilnaduensis* SZMC 22226 ^T^	1	0.125	0.125	8	0.03	0.06	0.25	2	0.25
*C. tamilnaduensis* SZMC 26758	0.5	0.125	0.125	16	0.03	0.25	0.25	2	0.125
*C. tamilnaduensis* SZMC 26759	1	0.125	0.125	16	0.25	0.25	0.25	2	0.25

^T^: type strain; AMB: amphotericin B; CLT: clotrimazole; ECN: econazole; FLC: fluconazole; ITC: itraconazole; KTC: ketoconazole; MCZ: miconazole; NTM: natamycin; TRB: terbinafine.
